# Brain-Derived Neurotrophic Factor: A Connecting Link Between Nutrition, Lifestyle, and Alzheimer’s Disease

**DOI:** 10.3389/fnins.2022.925991

**Published:** 2022-05-25

**Authors:** Bin Xue, Shah Mohammad Abbas Waseem, Zhixin Zhu, Mohammed A. Alshahrani, Nazia Nazam, Farah Anjum, Alaa Hamed Habib, Misbahuddin M. Rafeeq, Fauzia Nazam, Monika Sharma

**Affiliations:** ^1^School of Engineering, Guangzhou College of Technology and Business, Guangzhou, China; ^2^Department of Physiology, Jawaharlal Nehru Medical College, Aligarh Muslim University, Aligarh, India; ^3^Department of Clinical Laboratory Sciences, Faculty of Applied Medical Sciences, Najran University, Najran, Saudi Arabia; ^4^Amity Institute of Molecular Medicine and Stem Cell Research, Amity University, Noida, India; ^5^Department of Clinical Laboratory Sciences, College of Applied Medical Sciences, Taif University, Taif, Saudi Arabia; ^6^Department of Physiology, Faculty of Medicine, King Abdulaziz University, Jeddah, Saudi Arabia; ^7^Department of Pharmacology, Faculty of Medicine in Rabigh, King Abdulaziz University, Jeddah, Saudi Arabia; ^8^Section of Psychology, Women’s College, Aligarh Muslim University, Aligarh, India; ^9^Department of Zoology, Aligarh Muslim University, Aligarh, India

**Keywords:** brain-derived neurotrophic factor, neuroinflammation, Alzheimer’s Disease, lifestyle, nutrition

## Abstract

Brain-derived neurotrophic factor (BDNF) involving tropomyosin kinase B and low affinity p75 neurotropin receptors is the most abundant and researched neurotropins in mammal’s brain. It is one of the potential targets for therapeutics in Alzheimer’s disease (AD) owing to its key role in synaptic plasticity. Low levels of BDNF are implicated in the pathophysiology of neurological diseases including AD. However, a healthy lifestyle, exercise, and dietary modifications are shown to positively influence insulin regulation in the brain, reduce inflammation, and up-regulate the levels of BDNF, and are thus expected to have roles in AD. In this review, the relationship between BDNF, mental health, and AD is discussed. Insights into the interrelationships between nutrition, lifestyle, and environment with BDNF and possible roles in AD are also provided in the review. The review sheds light on the possible new therapeutic targets in neurodegenerative diseases.

## Introduction

A feature of multifactorial and heterogeneous neurodegenerative (Alzheimer’s disease; AD) is the self-association of neurotoxic β-amyloid (Aβ) oligomers forming Aβ monomers, which is indicative of abnormal protein processing. AD is the most prevalent (70%) dementia in the western world and is also becoming prevalent in developing countries and thus is a matter of public health concern (Qiu et al., 2007). As per WHO report of 2019, AD is the seventh leading cause of mortality worldwide and by 2050 around 106 million of individuals are expected to be suffering from its worldwide (Brookmeyer et al., 2007). Primitive preclinical AD, Mild cognitive impairment (MCI), and dementia are three stages of AD progression as per researchers (Albert et al., 2011; Jack et al., 2011; McKhann et al., 2011; Sperling et al., 2011). Hypertension; metabolic disorders (lipid profile derangements, diabetes, high BMI, and obesity) are implicated in the pathophysiology of AD. Other risk factors include genetics (APOEε4 allele &Val66Met), positive family history, gender, education, and previous history of head trauma (Bendlin et al., 2010; Xu et al., 2011). Apart from the above, metabolic and nutritional factors are also found to be associated with neurological disorders like parkinsonism, seizures, autism and AD (Napoli et al., 2014; Paoli et al., 2014) and thus they all require extensive research with special emphasis on finding and developing therapeutic targets to halt the disease progression and/or provide cure. AD is believed to affect the internal and external functioning of the brain by influencing the physiological processes (Chen et al., 2021). Brain-derived neurotrophic factor (BDNF) is the most abundant and well-studied neurotropin in mammalian brains. Because of its important role in synaptic plasticity, it is one of the potential therapeutic targets in AD. Low BDNF levels have been linked to the pathophysiology of neurological diseases such as AD (Miranda et al., 2019). A healthy lifestyle, exercise, and dietary changes have been shown to positively up-regulate BDNF levels, and are thus expected to play roles in AD (Sleiman et al., 2016; Colucci-D’Amato et al., 2020). The aim of the review article is to provide interaction among nutritional management, lifestyle modifications and BDNF levels in AD patients and look into possible mechanisms/pathways by which these interactions may halt the progression or prevent AD at early stages of life.

## Brain-Derived Neurotrophic Factor

The structure of human BDNF is very similar to that of mice and rats. It has an important role in maintaining synaptic plasticity and aids in memory storage functions of the hippocampus. BDNF signaling pathways regulates the expression of genes by activating the transcription factors CREB and CREB-binding protein (CBP), which in turn encodes proteins involved in brain plasticity, tolerance to stress and cell survival (Mattson et al., 2004). Plasma BDNF levels provide estimates of BDNF levels in the hippocampus and prefrontal cortex, and thus serve as a blood biomarker in AD patients (Klein et al., 2011).

Brain-derived neurotrophic factor binds to the tyrosine kinase receptor B (TrkB) and the non-specific p75 neurotrophin receptor (p75NTR). Dementia owing to hippocampal long-term potentiation (LTP) degradation appears as a consequence of a decline in BDNF levels or a defect in binding as demonstrated in various researches using BDNF and/or TrkB knockout animals (Chen et al., 1999; Kemppainen et al., 2012). Exogenous BDNF, on the other hand, facilitated LTP in BDNF- knockout animals (Patterson et al., 1996), thus providing evidence of a possible role of BDNF in AD. BDNF appears to have a role in cell survival and apoptosis by activating TrkB and p75NTR, respectively (Sandhya et al., 2013). BDNF also has an important roles in gene transcription regulation [NF-κB and c-Jun N-terminal kinase-p53-Bax (JNK)], intracellular signaling cascade activation [Ras/MAPK, PI3K/Akt, and phospholipase C through TrkB receptor activation], and neurogenesis (Diniz and Teixeira, 2011). In AD, the expression of BDNF are negatively influenced by the accumulation of Aβ amyloid (Meng et al., 2013; Zussy et al., 2013), thus furthering the possible role of BDNF and its signaling pathways in AD.

The impact of various factors and the overall regulation of BDNF is researched with emphasis on their role in NDs and memory functions (Erickson et al., 2012a). Because BDNF is linked to the Val66Met polymorphism (Xia et al., 2019) and is known to play a role in a variety of NDs, including Parkinson’s disease (PD) (Wang et al., 2019), and AD (Franzmeier et al., 2021). It must be thoroughly researched, with an emphasis on its therapeutic and preventive potential, in order to treat major depression disorder (MDD) and others.

## Advancing Age, Alzheimer’s Disease, and BDNF: Triad of Inconvenience

Advancing age is a risk factor for AD and on the other hand, BDNF levels are influenced by aging (Gaitán et al., 2021). Thus, there appears a common link between aging, AD, and BDNF which requires further exploration and extensive research. Cognitive impairment with advancing age is associated with reduced expression of BDNF and results of various studies have reported reduced concentrations in aged rodents, primates, and humans. Impairment may be correlated to defects in transcription, processing, and translation, as shown in studies conducted on aged rodents (Silhol et al., 2005; Shimada et al., 2014). Ethnicity and country of origin (White et al., 1996) decrease neuronal processing at the level of synapse (Burke and Barnes, 2006), and gray matter shrinkage with advancing age (Driscoll et al., 2009) appears to be important contributory factors as well.

## Lifestyle, Diet, Drugs, and BDNF Levels

Lifestyle, exercise, diet, and environmental factors also appear to influence BDNF signaling and thus appear to play a role in AD (Pistollato et al., 2018). High prevalence in the geriatrics population of African American and Japanese origin points toward the role of lifestyle as a risk/contributory factor in AD (Barnes, 2022). Animal models have reported that chronic stress and sedentary lifestyle negatively influence BDNF signaling (Perna and Brown, 2013; Vassoler et al., 2013). Flavanol, omega-3 fatty acids (FAs), cocoa polyphenolic, and apigenin have promising effects on BDNF levels (Cimini et al., 2013; Hjorth et al., 2013). Donepezil and galantamine (Acetylcholinesterase inhibitor) treated animal and human patients have shown to have higher levels of BDNF (Sakr et al., 2014). Aerobic exercises not only improves cognitive function but also minimizes the synaptic dysregulations (Baker et al., 2010; Intlekofer and Cotman, 2013). Thus, an exercise, diet, lifestyle, and drug centric models may be developed to provide better insights into the role of BDNF and its possible mechanisms and association with cognitive decline and AD. Effect of BDNF levels in different neurological conditions has been summarized in Table 1.

**TABLE 1:** Effects of BDNF (biomarker) levels in various neuropsychiatric conditions.

Neurodegenerative conditions	BDNF levels (Biomarker)	References
Alzheimer’s Disease	High levels of oligomeric Aβ in AD-related to reduced hippocampal and cognitive functions. Lower serum BDNF levels correlated with developing dementia followed by AD. Aβ lowered BDNF by reducing phosphorylated CREB protein.	Buchman et al., 2016; Amidfar et al., 2020
Major Depressive Disorder	Hippocampal slices showed decreased BDNF/Trk mRNA in MDD. Lower levels of BDNF are converted into higher levels using antidepressive drugs.	Matrisciano et al., 2009; Emon et al., 2020
Bipolar Disorder	Decreased serum or plasma levels of BDNF in the hippocampus of suicidal BD and altered DNA methylation in any stage (euthymic, depressive & manic).	Polyakova et al., 2015; Schröter et al., 2020
Parkinson’s disease	Lower levels of BDNF in serum associated with degeneration of the striatum in PD. The decreased expression is also connected with motor symptoms. Cognitive functions were also affected.	Scalzo et al., 2010; Palasz et al., 2020
Schizophrenia	Changes in BDNF levels in serum in SCZ patients. Decreased expression of BDNF/Trk mRNA in the dorsolateral prefrontal cortex and hippocampus in SCZ.	Rizos et al., 2008
Epilepsy	Epileptic seizures enhanced the levels of BDNF due to glutamate signaling. Elevated levels of BDNF mRNA expression in the hippocampus and cortex of the temporal lobe in epilepsy patients.	Martínez-Levy et al., 2018; Lin et al., 2020

## BDNF and Alzheimer’s Disease

Various studies have explored the potential roles of BDNF in etiopathogenesis and progression of NDs (Rosa et al., 2016; Eyileten et al., 2021). Studies have shown possible relationships between altered blood and CNS levels of BDNF and NDs including AD (Eyileten et al., 2021). Memory and cognitive defects have been associated with deregulation of BDNF, m RNA and protein (Rosa et al., 2016). The importance of BDNF in preventing neurodegeneration in AD has also been highlighted (Laske et al., 2011) by providing neurotrophic support (Ventriglia et al., 2013). Administration of BDNF improves cognitive dysfunctions, restores cell signaling mechanisms, and enhances expression of age related genes in animal models (Nagahara et al., 2009). Thus, high levels of BDNF are associated with lower risk of cognitive impairment in AD patients (Laske et al., 2011). The decline in BDNF levels is linked to increasing age, and it is more noticeable in females, the elderly, and those with higher body weights (Komulainen et al., 2008; Lee et al., 2009). The decline correlates with memory loss and hippocampal atrophy (Erickson et al., 2010).

Increased pro-inflammatory activity (Diniz et al., 2010), elevated oxidative stress and mitochondrial dysfunction (Swerdlow et al., 2010), diminished hippocampal neurogenesis (Shruster et al., 2010), and GSK3B hyperactivity (Forlenza et al., 2011) also play important roles in NDs. Because many of these mechanisms are regulated or influenced by BDNF and share intracellular regulatory pathways, understanding them is crucial. Thus, dysregulation in any one is going to have implications on others owing to interconnections and common regulations. On the other hand, improvement in any one is expected to have positive influences on other pathways. A pathological trigger can lead to a chain reaction that is expected to stimulate a cascade ultimately culminating in neurodegeneration and associated diseases like AD. Simply put, there appears to be a fine balance of pro- and anti-neurodegenerative pathways that is precisely regulated, and any change in homeostasis shifts the balance toward disease process (Swerdlow, 2007).

## Linkage of Lifestyle and Alzheimer’s Disease *via* BDNF

Brain-derived neurotrophic factor is the neurotrophin that is most susceptible to lifestyle changes. As demonstrated in animal studies, exercise and dietary balance appear to normalize BDNF levels, which have been reduced due to a high fat diet (Molteni et al., 2004). Regular exercise reduces the risk of developing dementia and AD after the age of 65 (Erickson et al., 2012b) which may be attributed to exercise-induced increases in BDNF and improved brain function, both of which influence cognitive functions positively (de Assis and de Almondes, 2017). Peripheral lactate and BDNF levels are shown to increase with high intensity exercises, whereas both central and peripheral levels of BDNF are upregulated with administration of lactate at rest. This is important as both lactate and BDNF levels are expected to stimulate neuroplasticity (Muller et al., 2020). Results have demonstrated enhanced remembrance and understanding of platform positions in Morris water maze, which were attributed to improvements in cognitive abilities in response to brief duration of exercise (Szuhany et al., 2015). When BDNF function in the hippocampus was inhibited in the former group, there was no difference in cognitive abilities. Running has been reported to increase neuronal spikes and improve spatial memory by increasing NTs (van Praag, 2008).

A single aerobic exercise session done consistently was associated with a higher increase in BDNF levels compared to if done acutely. A meta-analysis revealed that the results varied by gender, with males experiencing a greater increase than females (Szuhany et al., 2015). Interestingly, the increase in BDNF has not been consistent with strength/resistance exercises (Huang et al., 2014b). Only short-term transient increases in BDNF levels returning to baseline values post exercise have also been reported (Knaepen et al., 2010). As a consequence of physical exercise the CaMKII is activated indirectly by an increase in intracellular calcium ions, which in turn results in phosphorylation of CRE binding protein due to activation of CaMKII. Other possible pathways for enhancing BDNF *via* physical activity include the reactive oxygen species pathways. The proposed mechanism is that when neurons produce ROS in response to PE, the ROS activate the CRE binding protein, resulting in BDNF transcription *via* CREB activation (51).

## Linkage of Nutrition and Alzheimer’s Disease *via* BDNF

Epigenetics does play an important role in influencing the physiological homeostatic mechanisms. Environmental factors modulating human physiology largely depend upon lifestyle and dietary habits (Nicolia et al., 2015). Dietary modification is reported to reduce cognitive decline and risk of AD (Luchsinger and Mayeux, 2004). Advancing age and metabolic factors were observed as independent risk factors for AD in various epidemiological and animal studies. Long-term influence of diet on cognition appears to be exerted *via* effects on gene expression and regulatory frameworks (Dauncey, 2012, 2013; Gomez-Pinilla and Tyagi, 2013), which in itself are complex and need further exploration. Influences may vary from effects on hormones, neurotransmitters, metabolism, cell membrane function, and sand synaptic plasticity. The physiology of regulation of feeding provides the insights in understanding the complexities and possible role of diet. The energy state is shown to influence a variety of hormones (insulin, thyroid) and factors (BDNF) which in turn influence the expression of genes thereby influencing the physiology of brain (Dauncey, 2014). Interestingly, higher consumption of plant-based food are linked to lower trimethylamine oxide as well as higher fecal short chain fatty acids, fiber degrading microbiota and gut biodiversity (De Filippis et al., 2016; Garcia-Mantrana et al., 2018).

Similarly, studies have found that diet rich in saturated fatty acids and simple carbohydrates negatively influences memory and increases the risk of AD (Jackson et al., 2016; Alles et al., 2019). On the other hand, Medi (Mediterranean) diet is shown to exert anti-inflammatory properties, enhance insulin and BDNF production, and thus appears to be beneficial in reducing the risk of AD (Abuznait et al., 2013; Miquel et al., 2018).

**FIGURE 1:**
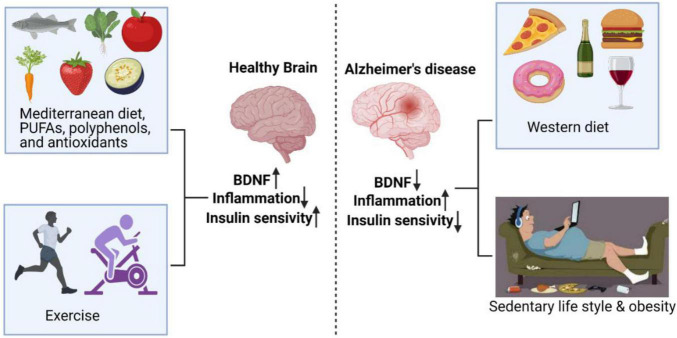
Exercise and diets rich in PUFAs, polyphenols, and antioxidants have positive effects on the brain, resulting in enhanced BDNF and insulin sensitivity, and decreased inflammation. On the other hand, sedentary behavior and Western diet enhanced AD risk by reducing BDNF and insulin sensitivity and increasing inflammation.

Thus, the combination of PE along with beneficial diet and/or dietary modification is proposed to reduce the risk/progression of AD. The combination is expected to be beneficial by virtue of enhanced anti-oxidants, polyphenols and polyunsaturated fat (PUFA) (Figure 1). PUFA (rich in long chain omega-3 FAs found in sea food) is expected to delay the memory decline and subsequent development of AD (Avallone et al., 2019). Polyphenols on the other hand exert beneficial effects *via* BDNF production, thereby positively influencing the learning and memory related areas of brain (Rendeiro et al., 2012; Tan et al., 2017). Omega 3 FAs exert anti-oxidant effects, maintain/normalize BDNF levels, and improve/increase learning ability following traumatic injury to brain (Avallone et al., 2019).

The role of life style and dietary modification (PE/CR/Exogenous BDNF) as futuristic treatment/interventions in AD appears appealing by virtue of the fact that both are non-invasive and easy to follow (but require self-control and determination).

## Other BDNF Inducers

Ginseng Radix extracts in cultured cortical cells are reported to stimulate the expression of BDNF *via* cAMP-response element-binding protein-dependent transcription (Fukuchi et al., 2019). Thus, BDNF appears a promising biomarker and therapeutic target for NDs. Recently, a pharmacologically validated library of 1280 compounds were tested for their ability to induce BDNF in neurons. Results were promising as Dipyrone (antipyretic drug) stimulated BDNF expression in neurons *via* Ca^2+^ influx (Fukuchi, 2020).

Flavonoids are shown to enhance spatial memory in animal models. Both factors increase BDNF expression by causing induction of Akt/PKB (Spencer, 2009; Bechara and Kelly, 2013). Thus, common links and pathways provide excellent therapeutic targets and thus warrant interventional studies. Many spices used traditionally almost in every household also have beneficial effects which need to be exploited for future interventions in NDs. One such an example is Curcumin. It is a promising candidate as it increases neurogenesis and BDNF by pathways involving inhibition of tau kinase JNK (Cole et al., 2007).

Studies with Mangosteen pericarp rich in xanthones (α-mangostin and γ-mangostin) in mice exhibit multiple effects ranging from increased BDNF levels in hippocampal slices, anti-inflammation and reduced tau levels, which ultimately provide neuroprotection and improvements in cognitive functions (Huang et al., 2014a). Findings are suggestive that a diet containing Mangosteen pericarp formulations can be used in AD patients to improve cognitive functions.

## Conclusion

Evidence-based research suggests that dietary and lifestyle changes can help people with AD. Calorie restriction has emerged as a potential means of preventing or delaying the onset of AD. Multiple complex pathways that may have both direct and indirect effects on brain physiology are linked to increased insulin secretion, anti-inflammatory effects, and, most importantly, BDNF production by the underlying protective mechanisms. Diet, exercise, and calorie restriction all seem promising because they are non-invasive and have the potential to influence the secretion of factors and hormones, making them candidates for the development of newer regimes in the management and prevention of cognitive impairments and memory loss. However, the beneficial effects may vary with the amount, duration, and intensity of modifications, which is especially true for exercise-induced changes. Thus, long-term prospective studies need to be designed to evaluate the effect of differential intensity of exercise on parameters influencing brain functions like cognition.

## Author Contributions

All authors listed have made a substantial, direct, and intellectual contribution to the work, and approved it for publication.

## Conflict of Interest

The authors declare that the research was conducted in the absence of any commercial or financial relationships that could be construed as a potential conflict of interest.

## Publisher’s Note

All claims expressed in this article are solely those of the authors and do not necessarily represent those of their affiliated organizations, or those of the publisher, the editors and the reviewers. Any product that may be evaluated in this article, or claim that may be made by its manufacturer, is not guaranteed or endorsed by the publisher.
